# Medication Management Initiatives Using Wearable Devices: Scoping Review

**DOI:** 10.2196/57652

**Published:** 2024-11-27

**Authors:** Haru Iino, Hayato Kizaki, Shungo Imai, Satoko Hori

**Affiliations:** 1 Division of Drug Informatics, Faculty of Pharmacy and Graduate School of Pharmaceutical Sciences, Keio University, Tokyo, Japan

**Keywords:** medication adherence, scoping review, database search, integrated medication management, drug, pharmacy, pharmacology, pharmacotherapy, pharmaceutics, medication, adherence, wearable, synthesis, review methods, digital health

## Abstract

**Background:**

Wearable devices (WDs) have evolved beyond simple fitness trackers to sophisticated health monitors capable of measuring vital signs, such as heart rate and blood oxygen levels. Their application in health care, particularly medication management, is an emerging field poised to significantly enhance patient adherence to treatment regimens. Despite their widespread use and increasing incorporation into clinical trials, a comprehensive review of WDs in terms of medication adherence has not been conducted.

**Objective:**

This study aimed to conduct a comprehensive scoping review to evaluate the impact of WDs on medication adherence across a variety of diseases, summarizing key research findings, outcomes, and challenges encountered.

**Methods:**

Adhering to PRISMA-ScR (Preferred Reporting Items for Systematic Reviews and Meta-Analyses Extension for Scoping Reviews) guidelines, a structured search was conducted across MEDLINE, Web of Science, and Embase databases, covering the literature from January 1, 2010, to September 30, 2022. The search strategy was based on terms related to WDs and medication adherence, specifically focusing on empirical studies to ensure the inclusion of original research findings. Studies were selected based on their relevance to medication adherence, usage of WDs in detecting medication-taking actions, and their role in integrated medication management systems.

**Results:**

We screened 657 articles and identified 18 articles. The identified studies demonstrated the diverse applications of WDs in enhancing medication adherence across diseases such as Parkinson disease, diabetes, and cardiovascular conditions. The geographical distribution and publication years of these studies indicate a growing interest in this research area. The studies were divided into three types: (1) studies reporting a correlation between data from WDs or their usage and medication adherence or drug usage as outcomes, (2) studies using WDs to detect the act of medication-taking itself, and (3) studies proposing an integrated medication management system that uses WDs in managing medication.

**Conclusions:**

WDs are increasingly being recognized for their potential to enhance medication management and adherence. This review underscores the need for further empirical research to validate the effectiveness of WDs in real-life settings and explore their use in predicting adherence based on activity rhythms and activities. Despite technological advancements, challenges remain regarding the integration of WDs into routine clinical practice. Future research should focus on leveraging the comprehensive data provided by WDs to develop personalized medication management strategies that can improve patient outcomes.

## Introduction

The term “medication adherence” refers to the extent to which patients correctly follow their medication regimens [1-4]. Medication adherence is influenced by lifestyle habits and the potential for using wearable devices (WDs) that record daily activities as activity data has been broadly considered [5-8].

WDs are currently equipped with various types of sensors [9-12]. Beyond standard heart rate or gyro sensors for activity and sleep tracking, some devices can measure or estimate blood pressure, blood glucose levels, blood oxygen levels, and various biomarkers [13-20]. Along with their widespread use, there has been an increasing trend in the use of these devices in health care. According to ClinicalTrials.gov, the number of clinical trials using WDs as outcome measures in drug-related interventions increased from 5, between January 1, 2016, and December 31, 2016, to 21 by 2021, marking a more than fourfold increase [21]. Due to their ability to provide long-term continuous monitoring with minimal burden on patients, there are reports of WDs being used not only in clinical trials but also in managing chronic diseases such as obesity, diabetes, and Parkinson disease, as well as in detecting conditions like COVID-19, cardiovascular diseases, and managing the side effects of chemotherapy [22-28].

In recent years, efforts have been made to use WDs to manage patient medication adherence. Medication management with WDs encompasses a wide range of approaches, including detecting medication-taking actions using motion sensors, using the device as an interface for information notifications, and analyzing various data recorded on the WDs to assess how accurately patients take their medications [29-31].

On the other hand, in the field of medication adherence, there has been a wide range of research methodologies and a lack of focus on specific disease areas, resulting in the absence of a review that provides research guidelines for the field. Therefore, this study seeks to conduct a comprehensive scoping review of WD applications across a spectrum of diseases, aiming to summarize key research findings, outcomes, and challenges encountered in medication management and adherence.

## Methods

### Databases and Search Strategy

The investigation was conducted in strict adherence to the PRISMA-ScR (Preferred Reporting Items for Systematic Reviews and Meta-Analyses Extension for Scoping Reviews) guidelines (Checklist 1) [32]. Overall, 3 major databases were searched: MEDLINE (through PubMed), Web of Science Core Collection (through Web of Science), and Embase (through ProQuest Dialog). Terms related to WDs and adherence were established separately for the search. These terms were appropriately divided into Medical Subject Headings (MeSH) terms and free words. For instance, in the MEDLINE search, the MeSH terms “wearable electronic devices” and “patient compliance” were used. Notably, under “patient compliance,” the inclusion of “medication adherence” was confirmed (Table 1). The search period was limited from January 1, 2010, to September 30, 2022. When filters were available, literature types such as reviews and editorial materials were excluded from the search.

**Table 1.** Search detail (MEDLINE).

Query number	Word	Search details
#1	Wearable electronic devices	“wearable electronic devices”[MeSH^a^ Terms] OR (“wearable”[All Fields] AND “electronic”[All Fields] AND “devices”[All Fields]) OR “wearable electronic devices”[All Fields]
#2	wearable devices	“wearable electronic devices”[MeSH Terms] OR (“wearable”[All Fields] AND “electronic”[All Fields] AND “devices”[All Fields]) OR “wearable electronic devices”[All Fields] OR (“wearable”[All Fields] AND “devices”[All Fields]) OR “wearable devices”[All Fields]
#3	“wearable device*”	“wearable device*”[All Fields]
#4	“smart wearable*”	“smart wearable*”[All Fields]
#5	“smart watch”	“smart watch”[All Fields]
#6	fitbit	“fitbit”[All Fields] OR “fitbits”[All Fields]
#7	“apple watch”	“apple watch”[All Fields]
#8	#1 OR #2 OR #3 OR #4 OR #5 OR #6 OR #7	#1 OR #2 OR #3 OR #4 OR #5 OR #6 OR #7
#9	Patient compliance	“patient compliance”[MeSH Terms] OR (“patient”[All Fields] AND “compliance”[All Fields]) OR “patient compliance”[All Fields]
#10	Medication compliance	“medication adherence”[MeSH Terms] OR (“medication”[All Fields] AND “adherence”[All Fields]) OR “medication adherence”[All Fields] OR (“medication”[All Fields] AND “compliance”[All Fields]) OR “medication compliance”[All Fields]
#11	#9 OR #10	#9 OR #10
#12	2010/01/01:2022/09/30 [Date - Publication]	2010/01/01:2022/09/30[Date - Publication]
#13	#8 AND #11 AND #12	#8 AND #11 AND #12
#14	review [Publication type]	“review”[Publication Type]
#15	systematic review [Publication type]	“systematic review”[Publication Type]
#16	#13 NOT (#14 OR #15)	#13 NOT (#14 OR #15)

a MeSH: Medical Subject Headings.

Database searches were conducted from November 2022 until December 2023. The search, removal of duplicates, and initial screening were performed by a single author (HI). Screening of potentially compatible references was independently performed by 2 authors (HI and HK). In case of any disagreements, the last author (SH) provided advice. The search process is shown in detail in Multimedia Appendix 1. Management of the literature and removal of duplicates were performed using Zotero (version 6.0.30; open-source software of the Corporation for Digital Scholarship).

### Inclusion and Exclusion Criteria for Papers

In this survey, no strict criteria were set for the research design or outcomes so as to encompass a broad range of studies that could potentially relate to the subject. However, each included study met at least one of the following conditions, which were also used to classify the study types in the Results section: (1) studies reporting a correlation between data from WDs or their usage and medication adherence or drug usage as outcomes, (2) studies using WDs to detect the act of medication-taking itself, and (3) studies proposing an integrated medication management system that uses WDs to manage medication. For condition 3, studies were only included if they clearly demonstrated the contribution of WDs to medication management, at least through notifications or other functions; studies that simply used WDs to obtain patient vitals or biometric information were excluded. Additionally, studies that conducted these types of investigations as a sub-analysis were also considered for inclusion in this review, even if it was not their main objective.

### Organizing the Results

Owing to the relatively small number of existing studies in this field, there is a wide variation in research methodologies, making it challenging to integrate the results into specific metrics. Therefore, in the Results section, we present which of the 3 study types outlined in the “Inclusion and Exclusion Criteria for Papers” section is satisfied by each selected paper. Furthermore, we provide insights into each study’s approach in the Discussion section. This scoping review aimed to provide an overview of the research field and not to analyze the effectiveness of interventions. Therefore, we did not critically assess the methodological quality of the included studies.

## Results

**Figure 1.**
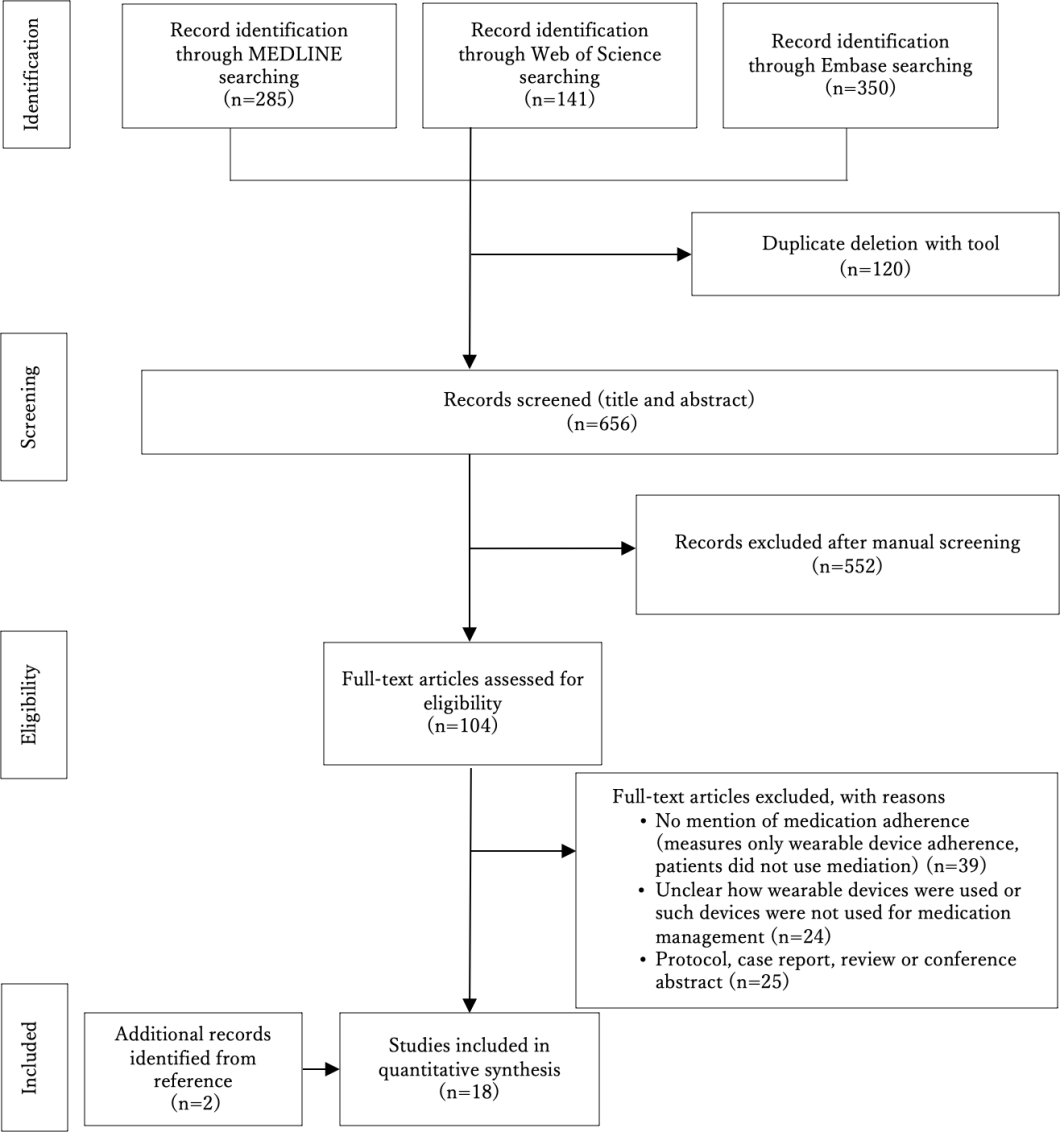
Flow diagram of study selection.

A flow diagram of the selection procedure is shown in Figure 1. Following the literature search and screening, we ultimately obtained 18 references. A summary of the literature is presented in Table 2.

**Table 2.** Summary of search results.

Author (published year)	Target population	Findings	Type of wearable device	Sample size, n	Usage of wearable devices	Country	Study type
Agurto et al (2021) [33]	Parkinson disease	It was possible to quantitatively determine the patient medication condition (on/off) due to the speed, acceleration, and symmetry of body movement.	Band (feet, wrist, lumbar, and sternum)	33	Record of acceleration	United States	1, 2
Quisel et al (2019) [31]	Hypertension, diabetes, and dyslipidemia	There is a significant relationship between medication adherence and activity tracking in individuals with chronic illness. Those who use activity tracking have higher medication adherence than those who do not use activity tracking.	Wristband	791‐10,499	Activity tracking (step count, sleep time)	United States	1
Zhang et al (2020) [34]	Hypertension	Wearable device nonusers have higher medication use scores (*P*<.001) On the other hand, medication use has a negative effect on adherence to the device use (no significant difference).	Wristband	317	Blood pressure monitoring	China	1
Zhang et al (2020) [35]	Hypertension	Wearable device nonusers have higher medication use scores (*P*=.003).	Wristband	212	Blood pressure monitoring	China	1
Cochran et al (2021) [36]	Patients with serious mental illness (major depressive disorder, schizophrenia, bipolar 1 disorder)	Higher values of both the previous day’s activity rhythm score and activity intensity score characteristics tended to be associated with higher next-day intake rates. Patients with mean activity rhythm scores greater than the patient-level median had higher overall intake rates than those with lower activity rhythm scores (*P*=.004).	Adhesive patch	113	Detection of medication intake, activity tracking (step counts, heart rates)	N/A^a^	1
Belknap et al (2013) [37]	Tuberculosis	The positive detection rate of medication behavior was 95% (95% CI 93.5‐96.2), and the specificity was 99.7% (95% CI 99.2‐99.9).	Adhesive patch	30	Detection of medication intake	United States	2
Browne et al (2019) [38]	Tuberculosis	The positive detection accuracy (percentage of correctly identified medications) of wirelessly observed therapy (WOT) was 99.3% (95% CI 98.1‐100). WOT medication adherence was noninferior to directly observed therapy (DOT) (WOT 95.6% vs DOT 92.7%; *P*=.31).	Adhesive patch	61	Detection of medication intake	United States	2, 3
Lee et al (2021) [39]	General patient (who commonly takes medication)	The medication behavior recognition by the developed model showed an accuracy of 92.7%, a precision of 0.909, and a recall of 0.949.	Wristband (camera attached)	89	Detection of medication intake	South Korea	2
Kalantarian et al (2016) [40][Bibr R35]	General population^b^	The constructed model was able to accurately classify chewable, saliva swallow, medicine capsules, conversation, and drinking water, with an average accuracy and recall of 90.17% and 88.9%, respectively.	Necklace	N/A^a^ (135 instances)	Detection of medication swallow using sensors	N/A	2
Fozoonmayeh et al (2020) [29]	General patient	*F*_1_-scores for the classification of medication activities (tablets, liquid agents) and nonmedication activities (sending SMS text messages, walking, writing, opening, and drinking bottle water) are up to 0.983.	Wristband	24	Tracking acceleration, heart rate, and atmosphere pressure	N/A	2
Spaulding et al (2019) [41]	After acute myocardial infarction	With the integration of the Corrie app and Apple Watch, the participants could be reminded to take drugs and track them directly on their wearable devices.	Wristband	60	Medication record, reminder (notification)^c^	United States	1, 3
da Silva et al (2019) [30]	Hypertension	Sending automatic reminders to patients via commercially available smartphones and smart TVs has reduced the number of missed medication doses and improved treatment compliance.	Wristband	N/A	Reminder (notification)	N/A	3
Levine et al (2019) [42]	Kidney or pancreas or liver transplant recipient (immunosuppressant user)	The use of mobile medical apps in this study did not indicate an increase in medication compliance.	Wristband	108	Reminder (notification)	United States	3
DiCarlo et al (2016) [43]	Hypertension	Positive detection accuracy (percentage of correctly identified medications) was 98% (95% CI 96.4-99.1).	Adhesive patch	37	Detection of medication intake	United Kingdom	2, 3
Noble et al (2016) [44]	Hypertension	Wearable device data revealed inappropriate drug use. Of 15 additional patients surveyed, 87% indicated that the device helped improve compliance.	Adhesive patch	54 (15 commercial pharmacies and 39 patients)	Detection of medication intake, activity tracking (rest, activity, and exercise)	United Kingdom	2, 3
Cochran et al (2022) [45][Bibr R45]	Schizophrenia	When categorizing engagement with the system as moderate and high engagement, based on medication adherence and device adherence as indicators, the average medication adherence rates were 0.62 for the moderate group and 0.87 for the high engagement group.	Adhesive patch	277	Detection of medication intake, activity tracking (rest, activity, and exercise)	United States	2, 3
Profit et al (2014) [46]	Patients with serious mental illness (major depressive disorder, schizophrenia, bipolar 1 disorder)	In the ingestion detection test, the detection rate of the ingestion sensor by the wearable sensor was 96.6%.	Adhesive patch	29	Detection of medication intake, Activity tracking (rest, activity, and exercise)	N/A	2, 3
Daar et al (2020) [47]	HIV	The system demonstrated its capability to collect real-time ingestion data and automatically send reminder SMS text messages to HIV patients undergoing ARV^d^ treatment.	Skin patch (adhesive)	15	Detection of medication	United States	2, 3

a N/A: not available.

b Although they were not patients, the study was conducted to simulate actual drug-taking behavior.

c The document only stated that “Medication adherence is also measured from the smartphone and smartwatch app usage data” and did not provide any details; however, we confirmed the functionality from an external site that provides an overview of the application.

d ARV: antiretroviral.

Among the selected literature, 2 (11.1%) papers were published before 2014, 8 (44.4%) papers between 2015 and 2019, and 8 (44.4%) papers between 2020 and 2022. Regarding the geographical distribution of the studies, North America accounted for 8 (44.4%, all from the United States) papers, Europe for 2 (11.1%, both from the United Kingdom) papers, and the Asia-Pacific region for 3 (16.7%, including 2 from China and one from South Korea) papers. However, 5 (27.8%) studies did not specify the study region. In terms of sample size, 6 (33.3%) papers had a sample size of 50 or fewer, 4 (22.2%) papers had between 51 and 100 participants, 2 (11.1%) papers had between 101 and 200 participants, 4 (22.2%) papers had over 200 participants, and 2 (11.1%) papers did not specify the sample size. Additionally, 3 (16.7%) papers did not specify a particular disease as their research subjects, whereas 15 (83.3%) papers focused on specific diseases. Among these, 4 papers addressed neuropsychiatric disorders, 6 (33.3%) addressed lifestyle-related diseases and 3 (16.7%) focused on infectious diseases.

We categorized each study into the following three study types: (1) studies reporting the relationship between WD data or device usage and medication adherence or drug usage as an outcome, (2) studies detecting medication-taking behavior directly using WDs, and (3) studies proposing integrated medication management systems where WDs are used. Below are examples of the study classifications.

### Study Type 1: Studies Reporting a Correlation Between Data From WDs or Their Usage and Medication Adherence or Drug Usage as Outcomes

Quisel et al [31] demonstrated that using WDs improved medication adherence among patients with hypertension, diabetes, and hyperlipidemia. Additionally, Cochran et al [36] showed a correlation between activity rhythm scores derived from WD data and medication adherence rates.

Studies classified under study type 1 investigate the relationship between medication adherence outcomes and the use or data of WDs, directly highlighting methodologies to clarify the involvement of WDs in medication adherence. These studies suggest the potential impact of WD use on adherence in real-world settings, as well as the relationship between WD-recorded data and medication adherence.

The studies suggest both positive and negative relationships between WD usage and medication adherence. However, the studies indicating a negative impact on adherence involved new WD distributions at the start of the research, not based on spontaneous usage. While one study suggests high adherence among a large cohort of spontaneous WD users, additional verification with other databases is needed. Studies using WD data reported that features derived from the data were correlated with medication adherence, suggesting the potential to estimate adherence from WD data.

### Study Type 2: Studies Using WDs to Detect the Act of Medication-Taking

Browne et al [38] demonstrated that a system combining ingestible sensors and WDs could detect medication-taking behavior with high accuracy. Kalantarian et al [40] developed a method using a necklace-type WD to detect medication-taking behavior through throat movements, achieving an accuracy of 90.17%.

Studies under study type 2 focus on detecting medication-taking behavior itself using WDs. Various methods for detecting medication-taking behavior, such as movement, video, and positional relationships with the medication, have been proposed and verified as practical attempts to contribute to the understanding of medication adherence.

Nearly all studies show that medication-taking behavior can be detected with high accuracy, often exceeding 90%. This is a necessary factor for real-world applicability. However, many of the devices used were proprietary, with only one study using a commercially available WD.

### Study Type 3: Studies Proposing an Integrated Medication Management System That Uses WDs to Manage Medication Adherence

Spaulding et al [41] demonstrated that a system combining a smartphone app and WD improved medication adherence in patients post-acute myocardial infarction. This system promoted regular medication intake through reminder functions, contributing to reduced readmission rates.

Studies under study type 3 do not focus solely on WDs but propose systems that manage medication adherence using IoT technology, with WDs serving as one of the interfaces. These studies discuss the development, specific usage, and verification results of medication adherence management systems using WDs, considering real-world applicability.

The studies indicate that WDs are useful interfaces for recording patient adherence. However, most studies used WDs primarily for notification or recording functions, without leveraging the multiple sensors embedded in WDs.

## Discussion

Initially, we describe studies that report WD data or associations with device use as an outcome of medication adherence or use (primarily those belonging to study type 1).

Agurto et al [33] investigated the impact of medication on physical activity levels in Parkinson disease patients. By objectively monitoring physical activity, they demonstrated that WDs can distinguish between medicated and non-medicated states. Parkinson disease, characterized by rapid and marked changes in physical activity following dopaminergic medication such as levodopa, is one area where WDs have been actively used [48-50]. However, this study is unique in that it extends the use of wearables to discern medication states.

Quisel et al [31] suggested that higher activity tracking and intensity using WDs are correlated with better medication adherence. Quisel et al [31] and Cochran et al [36] are among the few that have investigated the relationship between activity intensity and adherence. Quisel et al analyzed insurance databases using the proportion of days covered as an adherence measure. However, the proportion of days covered, by only calculating the prescribed days for medication, fails to confirm actual intake, thereby missing instances of nonadherence even when medication is prescribed [51,52]. Another limitation was the exclusion of medication purchases under different insurance plans that were not recorded in the database [31]. These limitations are difficult to overcome in database-based studies, and conducting validation using a different database is necessary to strengthen the results.

Cochran et al [36] scored individuals’ activity rhythms or patterns using WD data to analyze their correlation with medication adherence. Although adherence to chronic diseases is expected to be closely related to lifestyle, no studies have objectively measured lifestyle rhythms and analyzed their correlation with medication. The Cochran study showed that more consistent activity rhythms predict better adherence. However, the study’s limitations include the use of only 7 days of data and unclear thresholds for activity rhythm categorization, suggesting that further validation is needed for broader application.

Zhang et al conducted 2 parallel studies in 2020 at the same location but with different inclusion criteria: one encompassing all participants and the other focusing on individuals aged over 60 [34,35]. Notably, these studies contrasted with those of Quisel et al, indicating higher medication use scores among individuals not using WDs. However, these studies distributed new devices for the survey, differing from the group that wore the devices voluntarily [31]. Additionally, they focused primarily on device adherence, with medication usage as a secondary inquiry, without mentioning wearable-driven medication management systems. Additionally, this study defined a composite compliance score related to hypertension and reported a positive relationship between the composite compliance score, blood pressure values, and device usage. However, it also reported a negative relationship between medication use and these indicators. It is unlikely that there is a negative correlation between medication use and blood pressure values or hypertension indicators, suggesting the possibility of overlooking important factors such as age.

In type 1 studies, the nature of the target population differs, and the results are contradictory, making it difficult to draw consistent conclusions about the relationship between WD usage and medication adherence at this point. Among populations that regularly use WDs, a positive relationship between the amount of data recorded by the device and medication adherence has been suggested in relatively large samples. Considering the results from populations where WDs were distributed, it is possible that the temperament of those who use WDs reflects their medication adherence, indicating that WD usage itself may not be a factor that improves medication adherence. On the other hand, if appropriate behavioral features are derived from WD data, it may be possible to capture an individual’s medication adherence status.

Next, we discussed studies that detected medication-taking behaviors (study type 2). Although the types of data and devices used in these studies vary, all have achieved high accuracy in detection, suggesting that the technical feasibility of medication detection has already been established or is achievable. Some studies have developed both hardware and software independently, while others have only developed software independently. Using custom hardware allows for the collection of more data and the creation of more accurate predictive models tailored to patient outcomes and conditions. However, the costs of development and the lower recognition of these devices could hinder their widespread adoption in the general market.

Noble et al, Belknap et al, Browne et al, DiCarlo et al, Cochran et al, Profit et al, and Daar et al used patch-type devices [37,38,43-47]. These devices detect medication intake using sensors attached to the medication that transmit a signal to a patch worn on the body. Unlike smart pill bottles or detection methods based on arm movements, these patches offer a highly robust method for measuring medication adherence. Among the 7 studies using patch-type devices, 4 specifically reported on the accuracy of medication detection, each achieving an accuracy rate exceeding 95% [37,38,43,46]. This method is highly beneficial for diseases such as tuberculosis and schizophrenia, where continuous medication is crucial; however, the need for a sensor and transmitter for each medication makes it potentially expensive, necessitating a cost-effectiveness analysis for chronic diseases such as hypertension, where immediate adherence is not critical.

Kalantarian et al [40] specifically targeted the action of swallowing to detect medication intake, using a novel approach focused on throat movements. This unique study, which focused on throat movements, recorded a high accuracy of 90.17%. This also suggests that combining this method with smart pill bottles could further improve the accuracy. However, this throat-movement-detecting device, which is not commonly used, may face practical barriers because of its appearance and comfort.

Fozoonmayeh et al [29] and Lee et al [39] conducted studies to detect medication-taking behavior through arm movements using widely available wristband devices. Fozoonmayeh et al [29] used a commercial smartwatch, which could lower the clinical application barrier owing to its price and availability. Recognizing medication-taking behavior based solely on arm movements is challenging because of similarities with other actions, such as drinking water. However, the *F*_1_-score in this study was 0.983, indicating a highly effective model. Conversely, Lee et al [39] attempted to enhance accuracy by equipping the device with a camera and combining motion detection with image recognition. Although the model’s accuracy was lower at 92.7% compared to that of Fozoonmayeh et al [29], image recognition might be necessary to accommodate a variety of medication forms beyond the pills.

Finally, we describe a study proposing an integrated medication management system that uses WDs as part of the system (study type 3). Overall, research in this study supports comprehensive medication management by providing user-friendly interfaces. The primary functions are notifications and reminders, indicating that the role of WDs in medication management is limited. On the other hand, Spaulding et al [41] made it possible to record medication intake directly from a WD display, indicating its role in logging medication. WDs, which provide the most immediate interface for software and systems, have the potential to enhance the efficiency of disease-management systems by expanding their functionality.

These medication management systems are predominantly software-based, and many studies have used commercially available devices [53-55]. However, research such as that by Daar et al [47] involves developing custom devices. Although commercially available devices offer the advantages of being affordable and easily accessible, they have predetermined specifications and data capabilities that may not accommodate all the necessary metrics for certain diseases [11,56]. For instance, Kalantarian et al [40] used a unique necklace-type device to detect the swallowing motion, a form not commonly found in the market. Furthermore, many biochemical markers cannot be measured using commercially available devices, suggesting the need for developing custom devices to realize specific disease management strategies.

### Limitations

The limitations of this study include (1) restriction to original articles written in English and published in scientific journals and (2) room for improvement in the categorization of the studies (study type). The field of medication adherence management using WDs is relatively new, and projects in this area are expected to start with small-scale validations. Such studies are not always written in English or published in international journals. During our survey, we screened numerous abstracts from relevant conference presentations and non-English literature that appeared pertinent. However, obtaining complete access to these sources is often challenging, making their inclusion in reviews impractical.

Additionally, the diverse nature of studies on adherence management using WDs led to the categorization of some overlapping research types. Although this can be helpful in understanding the characteristics of the research, it is often duplicative and not a clear-cut classification. While we believe that this classification has minimal impact on the interpretation of the results in this review, defining clearer research directions as the field evolves could potentially facilitate a better understanding of prior studies.

### Future Directions

Further research is needed in various regions to understand the relationship between WD usage and medication adherence. On the other hand, the individual data from WDs can objectively capture patient behavior and create features related to adherence, such as daily rhythms and body movements influenced by medication. Currently, there are no reported medication management systems that fully use the various sensors and data WDs possess. However, in the future, by combining the detection of medication-taking actions, it is expected that WD data can be used to estimate individual medication adherence with high accuracy. Exploring better features that can be applied in real-world settings is crucial.

In the studies classified under study type 1 or 2, few have reached the point where they can be used by the general public as commercial products. As mentioned earlier, these studies face many gaps, including the need for basic theory validation, the development of consumer-oriented devices, and cost-effectiveness analysis. Addressing these research gaps with the aim of clinical application is expected to promote the use of WDs and improve medication adherence and patient health outcomes.

### Conclusions

Medication management using WDs is currently being implemented based on empirical research, primarily as a simple interface for patient notifications. Technically, the detection of medication-taking behavior has achieved high accuracy, necessitating real-life empirical studies to further leverage this technology. Notably, it has been suggested that specific medication-taking behaviors and daily activity rhythms are related to medication adherence [36]. These findings imply that WDs can predict patients’ medication adherence through daily activities and not merely by recognizing isolated medication-taking events. Exploring behavioral data from WDs in future research to clarify the relationship between patient lifestyles and medication practices promises to greatly expand the use of WDs in managing medication adherence.

## Supplementary material

10.2196/57652 Multimedia Appendix 1 Search strategy.

10.2196/57652 Checklist 1 PRISMA-ScR (Preferred Reporting Items for Systematic Reviews and Meta-Analyses Extension for Scoping Reviews) checklist.

## Abbreviations

MeSH: Medical Subject Headings
PRISMA: Preferred Reporting Items for Systematic Reviews and Meta-Analyses Extension for Scoping Reviews
WD: wearable device
