# Supplementary material for: Medication Management Initiatives Using Wearable Devices: Scoping Review
Source: JMIR Hum Factors. 2024 Nov 27;11:e57652. doi: 10.2196/57652 (PMC11612519; doi:10.2196/57652)
Supplement: Multimedia Appendix 1 [file humanfactors-v11-e57652-s001.docx]

Supplemental information

S2 **Searching strategy.**

S2-1 MEDLINE

| Query number | Word | Search Details | Results |
| --- | --- | --- | --- |
| #1 | Wearable Electronic Devices | "wearable electronic devices"[MeSH Terms] OR ("wearable"[All Fields] AND "electronic"[All Fields] AND "devices"[All Fields]) OR "wearable electronic devices"[All Fields] | 21,686 |
| #2 | wearable devices | "wearable electronic devices"[MeSH Terms] OR ("wearable"[All Fields] AND "electronic"[All Fields] AND "devices"[All Fields]) OR "wearable electronic devices"[All Fields] OR ("wearable"[All Fields] AND "devices"[All Fields]) OR "wearable devices"[All Fields] | 27,536 |
| #3 | "wearable device*" | "wearable device*"[All Fields] | 6,422 |
| #4 | "smart wearable*" | "smart wearable*"[All Fields] | 490 |
| #5 | "smart watch" | "smart watch"[All Fields] | 132 |
| #6 | fitbit | "fitbit"[All Fields] OR "fitbits"[All Fields] | 1,400 |
| #7 | "apple watch" | "apple watch"[All Fields] | 304 |
| #8 | #1 OR #2 OR #3 OR #4 OR #5 OR #6 OR #7 |  | 29,507 |
| #9 | Patient Compliance | "patient compliance"[MeSH Terms] OR ("patient"[All Fields] AND "compliance"[All Fields]) OR "patient compliance"[All Fields] | 124,593 |
| #10 | medication compliance | "medication adherence"[MeSH Terms] OR ("medication"[All Fields] AND "adherence"[All Fields]) OR "medication adherence"[All Fields] OR ("medication"[All Fields] AND "compliance"[All Fields]) OR "medication compliance"[All Fields] | 54,347 |
| #11 | #9 OR #10 |  | 142,189 |
| #12 | 2010/01/01:2022/09/30[Date - Publication] | 2010/01/01:2022/09/30[Date - Publication] | 14,838,226 |
| #13 | #8 AND #11 AND #12 |  | 336 |
| #14 | review[Publication Type] | "review"[Publication Type] | 3,197,759 |
| #15 | systematic review[Publication Type] | "systematic review"[Publication Type] | 235,027 |
| #16 | #13 NOT (#14 OR #15) |  | 285 |

#16 Full Query

((("wearable electronic devices"[MeSH Terms] OR ("wearable"[All Fields] AND "electronic"[All Fields] AND "devices"[All Fields]) OR "wearable electronic devices"[All Fields] OR ("wearable electronic devices"[MeSH Terms] OR ("wearable"[All Fields] AND "electronic"[All Fields] AND "devices"[All Fields]) OR "wearable electronic devices"[All Fields] OR ("wearable"[All Fields] AND "devices"[All Fields]) OR "wearable devices"[All Fields]) OR "wearable device*"[All Fields] OR "smart wearable*"[All Fields] OR "smart watch"[All Fields] OR ("fitbit"[All Fields] OR "fitbits"[All Fields]) OR "apple watch"[All Fields]) AND ("patient compliance"[MeSH Terms] OR ("patient"[All Fields] AND "compliance"[All Fields]) OR "patient compliance"[All Fields] OR ("medication adherence"[MeSH Terms] OR ("medication"[All Fields] AND "adherence"[All Fields]) OR "medication adherence"[All Fields] OR ("medication"[All Fields] AND "compliance"[All Fields]) OR "medication compliance"[All Fields])) AND 2010/01/01:2022/09/30[Date - Publication]) NOT "review"[Publication Type]) NOT "systematic review"[Publication Type]

S2-2 Web of Science Core Collection

| Query number | Query | Results |
| --- | --- | --- |
| #1 | ALL=(wearable devices) | 44044 |
| #2 | ALL=(Wearable Electronic Devices) | 17057 |
| #3 | ALL=("wearable device*") | 18115 |
| #4 | ALL=("smart wearable*") | 1778 |
| #5 | ALL=("smart watch") | 580 |
| #6 | ALL=(fitbit) | 1582 |
| #7 | ALL=("apple watch") | 410 |
| #8 | #1 OR #2 OR #3 OR #4 OR #5 OR #6 OR #7 | 46475 |
| #9 | ALL=(Patient Compliance) | 74452 |
| #10 | ALL=(medication compliance) | 14131 |
| #11 | #9 OR #10 | 76874 |
| #12 | #11 AND #8 | 199 |
| #13 | #12 AND タイムスパン: 2010-01-01 to 2022-09-30 | 160 |
| #14 | #13 NOT DT=(Review OR Editorial Material) | 141 |

S2-3 EMBASE

| Search number | Query | Results |
| --- | --- | --- |
| S1 | EMB.EXACT.EXPLODE("wearable computer") or "Wearable Electronic Devices" | 9923 |
| S2 | EMB.EXACT.EXPLODE("wearable device") OR "wearable devices" | 16861 |
| S3 | wearable device* | 7731 |
| S4 | smart wearable* | 507 |
| S5 | smart watch | 933 |
| S6 | fitbit or fitbits | 2329 |
| S7 | EMB.EXACT.EXPLODE("apple watch") or "apple watch" | 540 |
| S8 | S7 OR S6 OR S5 OR S4 OR S3 OR S2 OR S1 | 20695 |
| S9 | EMB.EXACT.EXPLODE("patient compliance") OR "Patient Compliance" | 206603 |
| S10 | EMB.EXACT.EXPLODE("medication compliance") OR "medication compliance" | 50778 |
| S11 | S10 OR S9 | 207108 |
| S12 | pd(20100101-20220930) | 19681172 |
| S13 | S12 AND S11 AND S8 | 435 |
| S14 | dtype(review) | 3201852 |
| S15 | EMB.EXACT.EXPLODE("systematic review") | 463645 |
| S16 | S13 not (S14 or S15) | 350 |
